# Resin-based sealing *vs* resin-modified glass-ionomer filling for treatment of occlusal cavitated dentine caries lesions in primary molars – a multi-center randomized controlled split-mouth 3-year follow-up clinical trial

**DOI:** 10.1007/s40368-026-01161-7

**Published:** 2026-01-10

**Authors:** Mats Bågesund, Ann-Christine Olson, Sharre Chizarie, Maja Omanovic, Anna Zuber, Ann Ingemansson Hultquist

**Affiliations:** 1 Department of Biomedical and Clinical Sciences (BKV), Linköping University, Linköping, Sweden; 2 Public Dental Service/Folktandvården Östergötland, Norrköping, Sweden; 3 Public Dental Service/Folktandvården Dalarna, Falun, Sweden

**Keywords:** Carious Dentin, Dental Sealant, Glass Ionomer Cement, Follow-up Study

## Abstract

**Purpose:**

To evaluate if covering occlusal cavitated dentine caries lesions with limited extension (no more than one third of the bucco-lingual or disto-mesial width of the occlusal surface) in primary molars with resin-based sealing (RBS)—without previous removal of caries—would result in a lower 3-year rate of success than if all caries was removed and the cavity was filled with a resin-modified glass-ionomer cement (RMGIC), and to assess if the experience of the two treatments differed according to evaluation performed by the children and the dentists.

**Methods:**

RBS and RMGIC treatments of occlusal cavitated dentine caries lesions with limited extension in primary molars were evaluated as “defective” or “defect-free” after 3, 6, 12, 24, and 36 months in a split-mouth-study of 66 children (30girls, 36 boys) aged 4.77 ± 1.27 years. The children ranked their own experience (C-Face) for each treatment on a 7-grade face-scale, and the dentists evaluated the child’s experience (D-C-exp), cooperation (D-coop) on a four-grade scale as either ‘very bad’, ‘quite bad’, ‘quite good’, or ‘very good’.

**Results:**

After three years 71.2% of RBS and 84.8% of RMGIC were defect-free (p = 0.093). D-C-exp was better for RBS (1.27 ± 0.51) than for RMGIC (1.53 ± 0.81) (p = 0.005), while no significant difference regarding C-Face or D-Coop was found between the two methods.

**Conclusion:**

RBS method for treatment of occlusal cavitated dentine caries lesions with limited extension (no more than one third of the bucco-lingual or disto-mesial width of the occlusal surface) in primary molars did not have a significantly lower rate of success, and was preferred by the patients, as compared to the conventional RMGIC method, according to the dentists’ evaluation of patient experience.

ClinicalTrials.gov Identifier: NCT05475145

## Introduction

A treatment need due to dental caries in a young child should be performed in a manner that will be as comfortable and safe as possible. Stressful, uncomfortable or painful procedures performed at an early age can contribute to the establishment of a long lasting dental anxiety or dental fear (Klingberg and Broberg 2007).

Several dental procedures for the treatment of dental caries include situations that may cause both discomfort and pain if they are not properly introduced with caution and professionalism—including the competence to meet the child with respect and empathy to build confidence and trust in the possibly stressful environment of the dental clinic (Gizani et al. 2022).The most stressful situations for dental patients are often the sight and feeling of the needle and the bur (Correa-Faria et al. 2020). Therefore, alternative treatment methods, where only some or no carious tissue is removed—such as: selective caries removal, stepwise caries removal, atraumatic restorative treatment, non-restorative cavity control, and sealing with stainless steel crowns (SSC) or resin- or glass ionomer-based sealing materials, have been used (Dorri et al. 2015; Correa-Faria et al. 2020; Schwendicke et al. 2021; BaniHani et al. 2022).

The ideal method for a sealing is pain-free, easy to perform, causing minimal discomfort to the patient, achieving a maximal esthetic result with an optimal fit, and a long-lasting isolation of the decayed part of the tooth from the surrounding oral cavity with tight closure.

Since Going et al. in 1978 found that sealing carious lesions using sealant material resulted in fewer bacteria and a decline in caries activity (Going et al. 1978), and since the study by Mertz-Fairhurst et al. in 1986 showed arrested caries (Mertz-Fairhurst et al. 1986), the sealing method has been used in several studies to avoid removal of healthy tooth structure and to avoid discomfort from dental treatment (Borges et al. 2012; Dorri et al. 2015; Alves et al. 2017; Qvist et al. 2017; Schwendicke et al. 2021; BaniHani et al. 2022), and has been found to be a possible treatment of occlusal cavitated dentine caries lesions (Qvist et al. 2017). The method has, anyhow, not yet been widely recognized as a reliable method of choice for clinical practice, and recent reviews have concluded that the scientific support for the sealing method has not been properly evaluated (Schwendicke et al. 2021; BaniHani et al. 2022). In a RCT study by Philip showing evident that sealing is an effective treatment approach to arrest the lesion of non-cavitated lesions on occlusal surfaces even if the lesion extends partly into the dentine (Philip & Suneja 2023).

*The aim* of this study was to answer the PICO-question: “In children, who had occlusal cavitated dentine caries lesions with limited extension (no more than one third of the width or the length of the occlusal surface) in two primary molars expected to exfoliate no earlier than three years ahead from the start of the study (P), does the RBS method without any previous removal of caries (I) result in a success rate (free from defects) that is lower, and in a better child experience (O) than when all caries was removed and a conventional RMGIC filling was placed in the prepared cavity (C), when evaluation of possible defects—defined as fractures, pits, secondary caries, loss of material or extraction related to occlusal caries or occlusal treatment (O) was performed during a 3-year follow up period, and is the experience among the children different when comparing the two methods according to a seven-grade face-scale and a four-grade dentist rating of child experience and cooperation (O)?”.

P: Children, who had occlusal cavitated dentine caries lesions with limited extension (no more than one third of the width or the length of the occlusal surface) in two primary molars expected to exfoliate no earlier than three years ahead from the start of the study

I: RBS method without any previous removal of caries compared to a conventional RMGIC filling preceded by injection and complete removal of dentine caries

C: Comparison of the experience according to the child and the dentist, and the success rate (absence of defects) of the two methods (RBS and RMGIC)

O: Experience during the treatment of the two methods according to a seven-grade face-scale and a four-grade dentist rating of child experience and cooperation. Evaluation during a 3-year follow up period of success rate – defined as absence of defects such as fractures, pits, secondary caries, loss of material or extraction related to occlusal caries or occlusal treatment.

*The hypotheses* were:

The RMGIC method has a higher share of success (defect-free without fractures, pits or loss of material, secondary caries or extractions related to occlusal caries or occlusal treatment) during the 3-year study than the RBS method.
a) Children and b) dentists rate the RBS method as better than the conventional RMGIC method for the treatment of occlusal cavitated dentine caries lesions in primary molars.

## Materials and methods

**Fig. 1:**
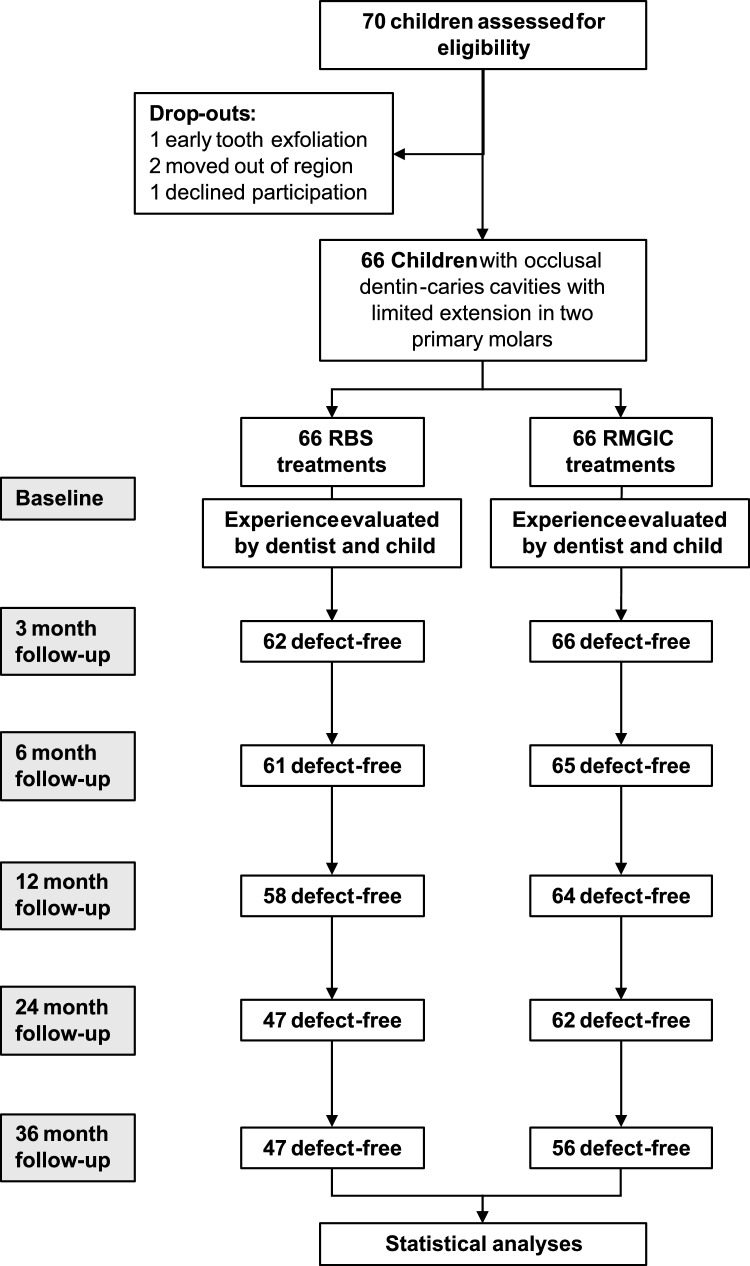
CONSORT flow diagram showing the flow of children and the follow up of the two treatment methods used in the split-mouth study

A CONSORT flow diagram of the study is presented in Fig. 1.

### Recruitment of patients

Seventy patients who fulfilled the criteria stated below were consecutively recruited to a randomized controlled study. Sixty-six children were followed up during the entire three-year follow-up period. Recruitment was conducted at six different clinics in the Public Dental Service, Östergötland County Council, Sweden.

### Inclusion criteria

- Primary occlusal cavitated dentine caries lesions with limited extension (involving no more than one third of the width or the length of the occlusal surface at a clinical examination with mirror and dental probe) in two primary molars was defined as a clinically diagnosed visual carious cavity that was not previously treated with filling or sealing, as determined by thorough visual examination and clinical evaluation with a dental probe of a dry primary molar occlusal surface (ICDAS 3-4).

### Exclusion criteria

- Children or parents/guardians unable to understand the information due to language problems or disabilities
- Less than 3 years until expected exfoliation of the decayed primary molar
- Hypomineralization interfering with the treatment area
- Previous filling or treatment of dentine caries on the occlusal or approximal surface of the primary molar
- The occlusal cavitated dentine caries lesion spreading over more than one third of the occlusal surface in one dimension (ICDAS 5–6)
- Children unable to cooperate during dental treatment because of age, language problems, disabilities, or medical disorders
- Approximal caries or filling – not originating from the originally included cavity—interfering with the occlusal RBS or RMGIC during the follow-up period
- Tooth exfoliated spontaneously before the end of the 36-month evaluation time
- Children leaving the public dental service or moving out of the region during the follow-up

A parent/guardian answered the Short Form of Children’s Fear Survey – parental version (CFSS-DS-pv) (Tollili et al. 2020)—including 15 questions are scored on a scale from 1 (not afraid at all) to 5 (very afraid)—before the start of the study.

From each participating child two primary molars with occlusal cavitated dentine caries lesions, that fulfilled the inclusion/exclusion criteria, were included in the study. If a child had more than two primary molars that fulfilled the inclusion criteria, a lottery was performed to decide which two teeth should be included in the study. The decayed teeth that were not included in the study were treated according to existing guidelines.

To avoid bias from possible changed cooperation and experience at the second treatment, unrestricted randomization was performed by drawing lots was performed for each child regarding which of the two treatment methods would be perform at the 1st and 2nd treatments. To avoid bias caused by a child’s possibly changed cooperation, experience and possible better handling by the dentist during the treatment of teeth at different positions in the mouth, another lottery was performed to determine which of the two teeth would be treated first.

The diagnosis of dentine caries and all treatments were performed by five experienced general dentists and one specialist in pediatric dentistry at six involved dental clinics.

No injection or caries removal but etching 20 s with 38% phosphoric acid of the cavity and the surrounding enamel followed by water spray and thorough air-flow drying preceded application of the light curing RBS. The RBS was performed with Delton opaque (Dentsply Sirona Pty Ltd, Mount Waverley, Australia) or Helioseal-F (Ivoclar Vivadent, Schaan, Liechtenstein) in 37 and 29 children, respectively, due to lack of availability. Light curing of the sealing material was performed with Light Emitting Diode (LED) light curing unit Planmeca Lumion Plus LED polymerisation light (Plandent OY, Helsinki, Finland) with output 1000 mW/cm^2^ for 20 s.

Topical (lidocaine 5% gel) and local anesthesia (Xylocain Dental Adrenalin 20 mg/ml + 12.5 µg/ml, Dentsply DeTrey GmbH or Septocaine 40 mg/ml + 55 µg/ml, Bigman AB) and complete caries removal with high-speed diamond bur (in some treatments supplemented with low-speed round bur) preceded the conventional filling with RMGIC (Fuji II LC, GC Company, Tokyo, Japan). Light curing of the RMGIC was performed with LED light curing units with output 1000 mW/cm^2^ for 40 s. If necessary, the filling was polished after the light curing. No additional treatment of the filling was performed. The methodology of the study followed a non-blinded randomized split-mouth design (Pozos-Guillen et al. 2017). The choice of the RMGIC method was used since it is found to be suitable for restoration of primary molars (Santos et al. 2016), and since the RMGIC method includes fewer steps (without etching and bonding) than composite techniques.

All treatments were performed without rubber dam, but with cotton roles, DryTips®, and saliva ejectors for moisture control.

The children ranked their own experience (C-Face) for each treatment on a 7-grade face-scale (Hicks et al. 2001).

The dentist evaluated the child’s experience (D-C-exp), cooperation (D-coop), and the overall experience of the treatment (D-exp) on a four-grade scale as either ‘very bad’, ‘quite bad’, ‘quite good’, or ‘very good’.

At the follow-up controls after 3, 6, 12, 24 and 36 months all the RBS and RMGIC, respectively, were evaluated as “defect-free” or “defect”, respectively, where’defect’ was defined as presence of pits, fractures, secondary caries, loss of (RBS or RMGIC) material, or extraction related to occlusal caries or occlusal treatment (RBS or RMGIC). ‘Defect-free’ was defined as absence of the mentioned defects. The classification was made only as ‘defect’ or ‘defect-free’, without specifying which subtype of defect was found. Extraction related to caries or treatment was recorded separately and as a defect. No calibration of the participating dentists was performed; however, the participating dentists all worked according to the same guidelines. The dentists diagnosed dentine caries (d3) using a visual tactile inspection with dental mirror and dental probe, according to the criteria by Koch (1967). Enamel caries was not registered. If a treatment was assessed as ‘defect’, the sealing or filling was repaired, but, thereafter, not included in further evaluations in the study.A power analysis revealed a significance level of 5% and a power of 95% when comparing the difference in success rates between the two treatment methods when 66 samples were included in each group (ClinCalc.com).

The children who fulfilled the inclusion criteria and who were not excluded due to the exclusion criteria underwent both one RBS- and one RMGIC-treatment each.

For statistical analyses—using the software SPSS^®^ (IBM, Armonk, NY, USA) Statistics 29—the McNemar-test was used to analyze the dependent, binary outcomes within the same patient, accounting for individual variations when comparing the two different treatment methods [McNemar 1947, Altman 1991], Wilcoxon-signed-rank test was used for evaluation of dentist´s and children’s total experience, paired t-test, and Spearman rank correlation analysis was used to evaluate these latter variables after 36 month. The significance level was set to 0.05.

**Fig. 2:**
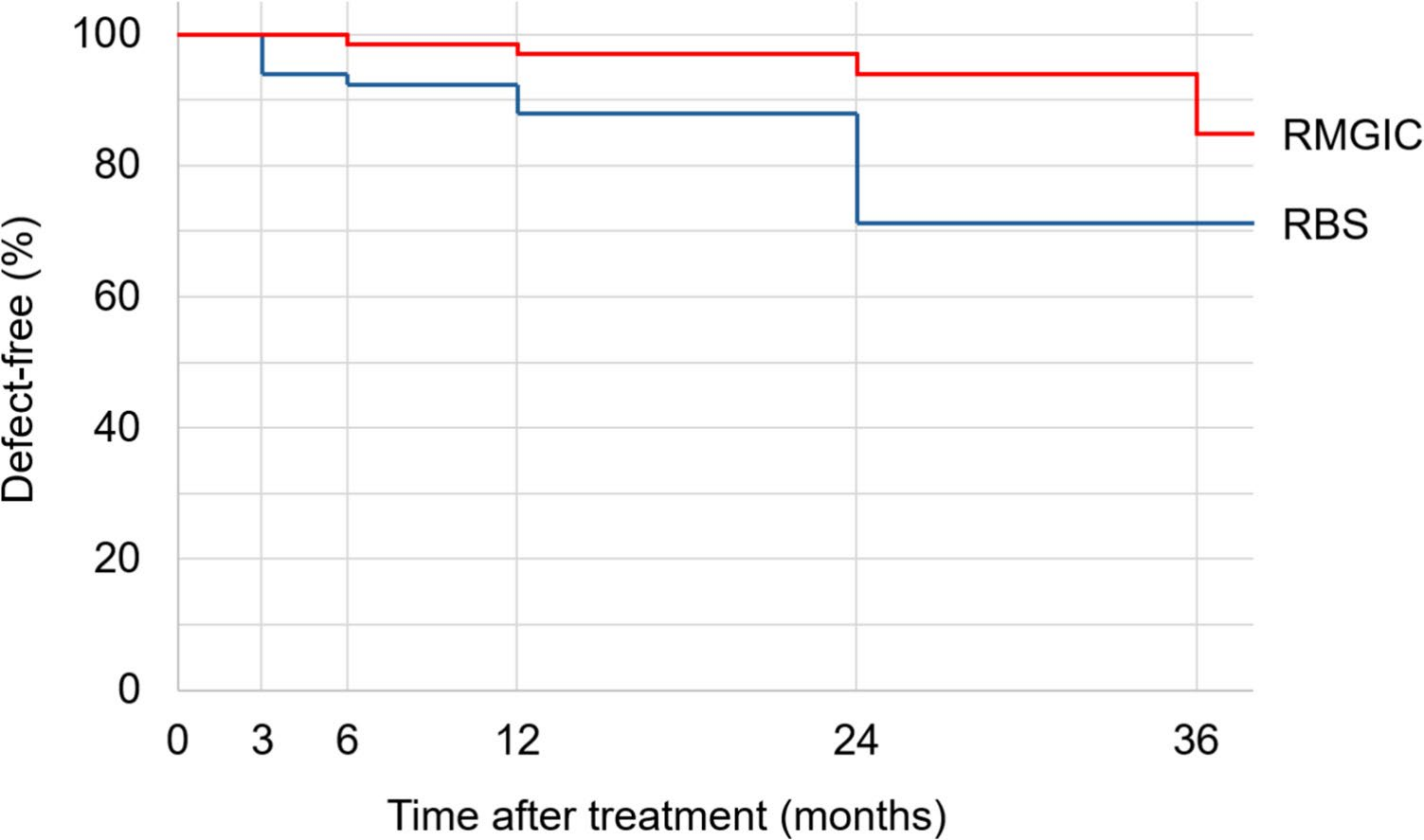
Survival curve describing the percentage of defect-free resin-modified glass-ionomer fillings (RMGIC) and resin-based sealings (RBS), respectively, during the 3-year follow-up (n = 66). Complete data and p-values are presented in Table 2

A survival analysis, describing the time when defects were found of the RBS and RMGIC, respectively, was performed and presented in a diagram as a survival curve in Fig. 2.

Ethical approval was obtained from the local ethical board before the start of the study.

Written informed consent to participate in the study was obtained from all participating children and both of their parents/legal guardians.

## Results

The baseline characteristics of the participating children are described in Table 1. The participating children had a mean ± SD [range] dmft of 3.89 ± 2.87 [1–13] at the age of 6 years.

**Table 1:** Baseline characteristics of the 66 participating children

	Boys (n = 36))	Girls (n = 30)	Total (n = 66)
	(Mean ± sd [Range])	(Mean ± sd [Range])	(Mean ± sd [Range])
Age (years)	4.59 ± 1.09 [2.9–8.5]	4.99 ± 1.45 [2.9–8.1]	4.77 ± 1.27 [2.9–8.5]
d	3.17 ± 1.99 [2–9]	3.23 ± 1.57 [2–7]	3.20 ± 1.80 [2–9]
e	0.22 ± 0.83 [0–4]	0.07 ± 0.25 [0–1]	0.15 ± 0.64 [0–4]
f	0.28 ± 0.85 [0–4]	0.70 ± 1.47 [0–6]	0.47 ± 1.18 [0–6]
deft	3.67 ± 2.35 [2–11]	4.00 ± 2.38 [2–12]	3.82 ± 2.53 [2–12]

d = dentine caries in primary teeth, e = primary teeth extracted due to caries, f = filled primary teeth

### Share of success

The success rate – describing the percentage of defect-free RMGIC- and RBS-treatments, respectively, during the entire 3-year study period—is shown in Fig. 2 and Table 2. After three years the accumulated share of success was 71.2% (47/66) for RBS and 84.8% (56/66) for RMGIC (p = 0.093), a non-significant difference according to the McNemar test. The significant difference (p = 0.0005) found between the methods after 2-years was no longer significant after 3 years (Table 2). Of the nineteen and ten treatments registered as defective among the RBS- and RMGIC-treated teeth, respectively, two of the treated teeth in each treatment group were extracted due to caries related pulpitis/periapical periodontitis – according to the dental records of the patients. But since the extracted teeth had previously been diagnosed with defects of the RBS- and RMGIC-treatments, these extractions were not included in the results. There were no additional defects of the RBS-sealings between the 2- and 3-year follow-up, while the number of new RMGIC defects continued to increase from 0 defects during the first 3 months to 6 new defects during the last year before the 3-year examination following RMGIC treatment.

**Table 2:** Comparison of the Resin-Based Sealant (RBS) vs Resin-Modified-Glass-IonomerCement (RMGIC) for treatment of occlusal cavitated dentine caries lesions with limited extension in primary molars during the 36 month follow-up (n = 66)

Accumulated share of success (Defect-free)			McNemar-test
Follow-up after	RBS % (n)	RMGIC % (n)	Binominal distribution p-value
3 months	93.9 (62)	100.0 (66)	0.25
6 months	92.4 (61)	98.5 (65)	0.125
12 months	87.9 (58)	97.0 (64)	0.070
24 months	71.2 (47)	93.9 (62)	0.0005
36 months	71.2 (47)	84.8 (56)	0.093

The accumulated success rate did not differ significantly between the two different RBS materials Delton opaque (27/37 = 73%) and Helioseal-F (20/29 = 69%) during the 3-year follow-up period.

### Experience and Cooperation

The child experience and cooperation is shown in Table 3. The D-exp for RBS (1.20 ± 0.54) was not significantly better (p = 0.076) than for RMGIC (1.38 ± 0.75), the D-C-exp was better for RBS (1.27 ± 0.51) than for RMGIC (1.53 ± 0.81) (p = 0.005), and the D-coop did not differ (p = 0.512) between the RBS (1.30 ± 0.58) and RMGIC (1.35 ± 0.71). The C-Face rating of the RBS method (1.47 ± 1.06) was not significantly (p = 0.21) better than RMGIC (1.79 ± 1.65).

**Table 3:** Comparison of the Resin-Based Sealant (RBS) vs Resin-Modified-Glass-Ionomer Cement (RMGIC) for treatment of occlusal cavitated dentine caries lesions with limited extension in primary molars during the 36-month follow-up (n = 66)

	RBS	RMGIC	p-value	statistical method
Dentist rating of total experience	1.20 ± 0.54	1.38 ± 0.75	0.076	WSR
Dentist rating of child experience	1.27 ± 0.51	1.53 ± 0.81	0.005	WSR
Dentist rating of child cooperation	1.30 ± 0.58	1.35 ± 0.71	0.512	WSR
Child experience (Face-scale)	1.47 ± 1.06	1.79 ± 1.65	0.209	WSR

WSR, Wilcoxon Signed Rank test

### 1st vs 2nd treatment

The accumulated defect rate was not significantly higher (p = 0.30) in the RBS group when the treatment was performed as the first treatment (15/46, 32.6%) than when RBS had been performed as the second treatment (4/20, 20.0%). The RMGIC group had almost the same defect rate whether treatment was performed as first (3/20, 15.0%) or second (7/46, 15.2%) treatment (p = 0.98).

### Drop out

Of the 70 children (34 girls, 36 boys) included in the study, two moved out of the region during the study period, and one did not turn up at the 3-year visit. Another child was excluded because one of the treated teeth had exfoliated before the end of the 3-year follow-up period.

### Study group

The remaining 66 children (30 girls, 36 boys) included in the 3 year follow-up study had a mean ± SD (min–max) age of 4.77 ± 1.27 (2.9–8.5) years, and 5.15 ± 1.29 (3.0–8.9) years, at the first and second treatment, respectively.

The mean CFSS-DS-pv score before the first treatment was 25.5 ± 8.3 (14–43) with seven (11%) of the children having a score ≥ 38, indicating severe dental fear (Klingberg et al. 1994; Dahlander et al. 2019).

The first treatment was performed with RMGIC in 20 (30.3%) of the children, and with RBS in 46 (69.7%) of the children.

### Complications

According to the patients’ dental records, four teeth with previously registered defects were extracted due to occlusal caries-related periapical periodontitis or pulpitis. Two of the RMGIC-treated teeth were extracted—one due to extensive caries after 28 months and one due to caries related periapical periodontitis with buccal abscess after 23 months. Two RBS-treated teeth were extracted after 29, and 35 months, respectively, both due to caries related pulpitis. No other serious adverse events or acute conditions related to the studied teeth were observed during the 3-year follow-up period.

### Correlation of variables

The Spearman correlation coefficients for RBS and RMGIC related to the evaluated variables are shown in Table 4. The accumulated share of RMGIC success after 36 months was correlated with D-exp, D-C-exp and D-coop. The accumulated share of RBS success after 36 months was not correlated with any of the studied variables.

**Table 4:** Spearman rank correlation (r_s_) for the evaluated variables during the 36-month follow-up of RBS and RMGIC, respectively

Variables (Rank levels)	RBS accumulated success rate after 36 months [r_s_ (p-value)]	RMGIC accumulated success rate after 36 months [r_s_ (p-value)]
D-exp (1–4)	0.089 (0.482)	0.376 (0.002)
D-C-exp (1–4)	0.038 (0.764)	0.258 (0.036)
D-coop (1–4)	0.026 (0.837)	0.352 (0.004)
C-Face-exp (1–7)	−0.094 (0.455)	0.239 (0.053)
CFSS-DS-pv (15–75)	0.094 (0.458)	0.106 (0.401)

RBS, Resin-Based Sealing; RMGIC, Resin-Modified-Glass-Ionomer-Cement filling; D-exp, Dentist-ranking of total experience of treatment; D-C-exp, Dentist-ranking of child experience during treatment; D-coop, Dentist-ranked cooperation; C-Face, Child-ranked experience on Face-scale; CFSS-DS-pv, Parental version of the Short Form of the Children’s Fear Survey

## Discussion

### Primary finding

The 3-year results from the present study revealed that treatment of limited occlusal cavitated dentine caries lesions in primary molars with conventional RMGIC fillings had fewer defects than when the RBS was used, and the RMGIC-method performed better than RBS after two years (Table 2). However, since the difference in the share of success was not significant after 3 years, and because defects complemented with further material did not cause any further complications, we suggest that RBS can be used for treatment of primary molars, when it is not possible to perform a conventional filling in a reasonable source of time, if the occlusal cavitated dentine caries lesion has a limited extension, and (only) if resources for frequent follow up are available.

After the start of the study, several reports have found that bonding before application of sealing material will increase the quality of resin-based sealings (Bagherian et al. 2016). Bonding was not applied prior to sealing in our study, and future studies including the application of bonding prior to sealing will probably increase the longevity of the RBS technique.

Several different studies have evaluated fissure sealants for treatment of dentine caries in primary molars, with similar results (Schwendicke et al. 2021).

Our finding that the majority of defects were found during the first part of the follow-up period is in accordance with a 36 month follow-up study of 30 RBS sealings, where RBS had a high defect rate after 18 months; however, in the later part of the study, only a few defects were observed (Borges et al. 2012).

Other studies with shorter follow-up periods have found significantly better outcomes with fillings than with sealings (Hesse et al. 2014; Qvist et al. 2017). The similar tendency towards lower success rate for the RBS in our study did not cause any severe problems, since all defective dental sealings and fillings were mended when the defects were noticed, and – with the exception of two previously ‘defect’ extracted teeth in each group—all teeth in the study could be followed until the 36 month follow-up visit.

There was a tendency towards a better outcome of the RMGIC method, but since the difference was not significant, the first hypothesis could not be accepted.

Every child with recent active dentine caries should be regarded as a patient at risk for the development of new carious lesions (Hultquist et al. 2021). Every child with an increased caries-risk should receive an individualized complementary caries preventive program and preferably visit the dental clinic for regular caries preventive measures (Rubin et al. 2019). At these visits, the existing fillings or sealings should be checked and, if necessary, mended.

It is generally recognized that the need for gentle dental treatment to minimize pain and discomfort is especially valuable for children (Dipalma et al. 2025). The results from the evaluations of child experience and cooperation, performed by the participating children and dentists in the present study, were expected, since non-invasive and minimal-invasive techniques are known to cause less pain, discomfort and anxiety than traditional treatment of cavities with rotating instruments (Dipalma et al. 2025).

The second hypothesis - that children and dentists rate the RBS method as better than the conventional RMGIC method for the treatment of occlusal cavitated dentine caries lesions in primary molars - was accepted.

The use of RBS has been suggested by others (Brignardello-Petersen 2019) as an option for the treatment of occlusal cavitated dentine caries lesions in primary molars, for example, in children with disabilities or when the child is not mature enough to accept difficult dental treatment, such as dental injection or rotary instruments. To postpone definite caries treatment a few years by using the RBS technique will reduce the risk of developing dental anxiety for many children, who are immature to cooperate with the use of injection or the dental bur, and be of great value for the individual child, not least in regard to possible future dental treatment needs (Klingberg and Broberg 2007). The present study did not include any comparisons to methods other than the RBS and RMGIC. Therefore, it is reasonable to consider that some patients would benefit more from other types of treatments, such as the Hall technique (Innes et al. 2015) or composite restorations. The Hall technique will include approximal surfaces, making it a better option when caries-active children are treated. The Hall technique is often (Innes et al. 2017), but not always (Bell et al. 2010), pain-free and is known to cause less discomfort for the patient than the conventional ‘drill-and-fill’ technique (Innes et al. 2015). Therefore, many dentists would benefit from learning to use the Hall technique more frequently, since the Hall technique is technically more difficult to perform (Innes et al. 2015; Narbutaite et al. 2024).

### Study design

The power analysis confirmed that the final sample size was sufficient to analyze the main (first) hypothesis.

Due to the split-mouth design (Pozos-Guillen et al. 2017) of the study, the bias from patient selection – such as patient background and different caries risk, was considered to be of minor importance for the analysis of the first and main hypothesis. The possibility that some groups of patients or families may have denied participation or were excluded because of the exclusion criteria should be considered when interpreting some of the results.

The accumulated success rate did not differ between the two RBS materials. Therefore, the use of two different RBS materials did not influence the results.

The children were ordinary patients at public dental clinics or referred for specialist dental care at a specialist clinic for pediatric dentistry. It could be discussed whether examination and treatment at a specialist clinic by a specialist in pediatric dentistry, would influence the performance of treatment and the experience and cooperation of the children. But since only five of the 66 included children had their treatment performed at the specialist clinic, we believe that the influence from different categories of personnel has not influence the results to any significant way. The CFSS-DS showed a similar mean score as in two previous Swedish studies in similar age groups (Klingberg et al. 1994; Dahlander et al. 2019) with a tendency towards slightly higher values in our present study (25 as compared to 23–25 in the previous studies) for both CFSS-DS and the percentage of children with a score of 38 or more (11% in our study and 7% in the previous studies), indicating that the children in our study had a distribution regarding dental fear similar to the previously mentioned studies (Klingberg et al. 1994; Dahlander et al. 2019). A higher score may contribute to cooperative problems, which should be considered when interpreting the results of our study.

A limitation of the study was that no calibration of the participating dentists was performed regarding the examination procedure, causing an increased risk of bias related to different evaluations performed by the examining dentists. However, since the dentists were trained and educated to work according to the same guidelines, and since the inclusion criteria described the size of the occlusal cavitated dentine caries lesions and the defects at the follow-up were described, we consider that this risk for bias was of minor importance for the results.

We did not consider it possible to mask for participants or care providers, since the methods were very different in performance and had different appearances in the mouth.

The choice not to include non-treated teeth with dentine caries as a control group was made because the risk for complications was considered too high and would not have been accepted due to ethical considerations. This decision was supported by findings in a previous similar study of permanent teeth by Borges et al. (2012), in which they had to exclude the entire control group of untreated teeth because of caries progression in almost all (25 of 26) of the teeth.

Due to logistical reasons, no sequence generation was performed to optimize randomization. In future studies, the use of sequence generation should be considered to optimize the randomization procedure.

The reason for the observed drift towards more frequent first treatment with RBS may indicate a bias in the randomization procedure. The easier RBS procedure may have been used as an introduction to the later performed RMGIC treatment, making the first treatment easier to perform in an immature or uncooperative child. Interviews with the dental personnel at the participating clinics indicated that sealing was sometimes performed when no other treatment was considered possible to perform. The circumstance that the first treatment was more frequently performed with the RBS method may have contributed to a longer treatment time for the RBS method, since the first treatment is expected to take a longer time due to a greater need for introduction of the child. These circumstances should be considered when interpreting the results of this study.

Rubber dam has been suggested during dental treatment, but was not used in this study, because rubber dam is seldom used by dentists for these procedures (Roulet 2010), and we wanted to have conditions relevant to the established conventional practice among dentists. Studies have shown varying results, with little evidence regarding the use of rubber dam or cotton rolls during such treatments (Keys and Carson 2017).

To minimize intervention, the method used for the RBS technique in the present study did not include any cleansing of the occlusal surface. This could be a reason why such a high defect rate was observed, since remnants of debris and the pellicle may have reduced the ability of the etching agent to reach the surface, thereby, causing a defect retention of the resin-based sealant (Bagherian and Sarraf Shirazi 2016).

According to the manufacturer’s recommendations, the etchant should have been kept in place for 30 s, but due to limited patient cooperation or patience, the time for etching was sometimes shortened, according to the participating dentists. This may have influenced the retention of the RBS on the enamel, since it has been found that a longer time for etching increases the retention of the sealant (Lo et al. 2019).

The reason for choosing the materials and methods for the study can be discussed. RBS has been found to have an equal caries-preventing effect as RMGIC sealant in permanent teeth (Mickenautsch and Yengopal 2016; Alirezaei et al. 2018). However, due to the limitations of the RMGIC, including lack of toughness, early water sensitivity, low abrasion resistance, and porosity, and especially the better retention found for RBS as compared to RMGIC sealant (Lenzi et al. 2016; Alirezaei et al. 2018), we preferred to use the RBS, since we found it of utmost importance to achieve a tight sealing of the cavity and, thereby, avoiding substrate leakage. The self-etch system has been shown to have a lower retention to enamel than the etch-and-rinse performance (Botton et al. 2016; Dias et al. 2018). Therefore, the etch-and-rinse system for RBS in the present study was considered a relevant choice.

The use of an RBS without the material related benefits of composites, such as resistance to wear and less risk of fractures, could be avoided by using a flowable composite instead of an RBS (Mickenautsch and Yengopal 2016). In a two-year follow-up study of 21 children performed by Dias et al. (Dias et al. 2018), it was stated that RBS over occlusal caries in primary molars showed equal lesion progression compared to partial caries removal followed by composite filling, indicating that a tight sealing is more important than removal of all caries. During the introduction of the participating dentists before the start of the study, they found it important to cover the entire occlusal surface with the etchant gel and to make the RBS placed over dental caries substantially thicker than when a fissure sealing is performed for caries prevention.

### Caries progression

The question of whether there were any active caries left below the RBS is relevant. Previous studies have shown that there is no difference in the progression of the caries process between RBS (without caries removal) and a flowable composite after partial caries removal (Hesse et al. 2014). To avoid progression of the caries lesion, it is important to optimize the cleansing of the surface and to optimize the conditions to obtain a tight sealing of the entire cavity to avoid leakage of substrate (carbohydrates) into the cavity (Hesse et al. 2014). In our study, where several of the children did not cooperate with intraoral radiographs and others had no individual indications for radiographs, we were not able to make any radiographic evaluations of possible caries progression, other than in a limited number of patients. The opinion among the participating dentists was that, according to the clinical and (when present) radiological results, no obvious progression of the dentine caries depth was found during the 36 months study period.

### Discomfort

The better experience of the RBS treatment as evaluated by both the children and the dentists, as shown in Table 3, is in agreement with findings from other studies (Innes et al. 2015; Santamaria et al. 2015).

Regarding the assessment of patient experience according to the 7-grade face-scale, it could be mentioned that several of the dentists involved noticed that the child often marked a happier (lower numbered) face than expected from the dentist according to the cooperation and behavior during the treatment. This could have been caused by the children wanting to be kind to the dentist, but at the same time providing bias to the evaluation of the study. This should be considered when interpreting these results. The use of a face-scale originally used for pain assessment could be discussed, since pain is a cause of discomfort, while discomfort not always includes pain, and since the experience of pain and discomfort is not easy to separate in younger children, this might have influenced the children’s answers.

We hope that the use of the described RBS treatment strategy for treatment of occlusal cavitated dentine caries lesions with limited extension in primary molars—will:

- enable postponement of extensive treatment in immature children until the child can cooperate in the dental setting.
- be a long-term temporary alternative to conventional dental treatment, including injection and removal of carious tissue in uncooperative children.

## Conclusion

The better performance of the RMGIC method found after 2 years was no longer significant after 3 years. The RBS method was preferred by the patients, according to the dentists’ evaluation of patient experience. If an occlusal cavitated dentine caries lesion in a primary molar has a limited extension (no more than one third of the bucco-lingual or disto-mesial width of the occlusal surface), the described RBS-method can be used, if it is not possible to perform a conventional filling in a reasonable source of time, and if resources for frequent follow up are available.

## Data Availability

This study was registered in the clinical trial registry at ClinicalTrials.gov (ClinicalTrials.gov Identifier: NCT05475145), where data are available in the document section.
